# Modulating environmental signals to reveal mechanisms and vulnerabilities of cancer persisters

**DOI:** 10.1126/sciadv.abi7711

**Published:** 2022-01-28

**Authors:** Xiaoxiao Sun, Jake M. Bieber, Heinz Hammerlindl, Robert J. Chalkley, Kathy H. Li, Alma L. Burlingame, Matthew P. Jacobson, Lani F. Wu, Steven J. Altschuler

**Affiliations:** Department of Pharmaceutical Chemistry, University of California, San Francisco, San Francisco, CA 94158, USA.

## Abstract

Cancer persister cells are able to survive otherwise lethal doses of drugs through nongenetic mechanisms, which can lead to cancer regrowth and drug resistance. The broad spectrum of molecular differences observed between persisters and their treatment-naïve counterparts makes it challenging to identify causal mechanisms underlying persistence. Here, we modulate environmental signals to identify cellular mechanisms that promote the emergence of persisters and to pinpoint actionable vulnerabilities that eliminate them. We found that interferon-γ (IFNγ) can induce a pro-persistence signal that can be specifically eliminated by inhibition of type I protein arginine methyltransferase (PRMT) (PRMTi). Mechanistic investigation revealed that signal transducer and activator of transcription 1 (STAT1) is a key component connecting IFNγ’s pro-persistence and PRMTi’s antipersistence effects, suggesting a previously unknown application of PRMTi to target persisters in settings with high STAT1 expression. Modulating environmental signals can accelerate the identification of mechanisms that promote and eliminate cancer persistence.

## Teaser

Modulating environmental signals accelerates the discovery of cancer persister mechanisms and vulnerabilities.

## INTRODUCTION

Persistence in cancer is defined functionally by the ability of cells to reversibly enter a state that enables them to survive presumed lethal concentrations of a drug (*1*–*4*). Persisters, the small fraction of cells that persist during drug treatment, are an impediment to durable efficacy of treatment as they can serve as “reservoirs” for the eventual emergence of acquired resistance (*2*, *5*). Mechanisms underlying the phenomena of persistence remain poorly understood. What signals promote the emergence of persisters during cancer drug treatment? What perturbations can synergize with the cancer drug to effectively eliminate persisters within the bulk drug-sensitive population? Much as with bacterial persisters (*6*–*9*), elucidating mechanisms of cancer persisters has been challenging due to their reliance on nongenetic, reversible mechanisms for survival and small subpopulation size. We hypothesized that extracellular factors, such as might be found in a tumor microenvironment, can help to find intracellular signal transducers that promote cancer persistence and provide functional assays for elucidating novel persister vulnerabilities.

## RESULTS

### Develop scalable assay and sensitive readout for persistent cancer cells

**Fig. 1.**
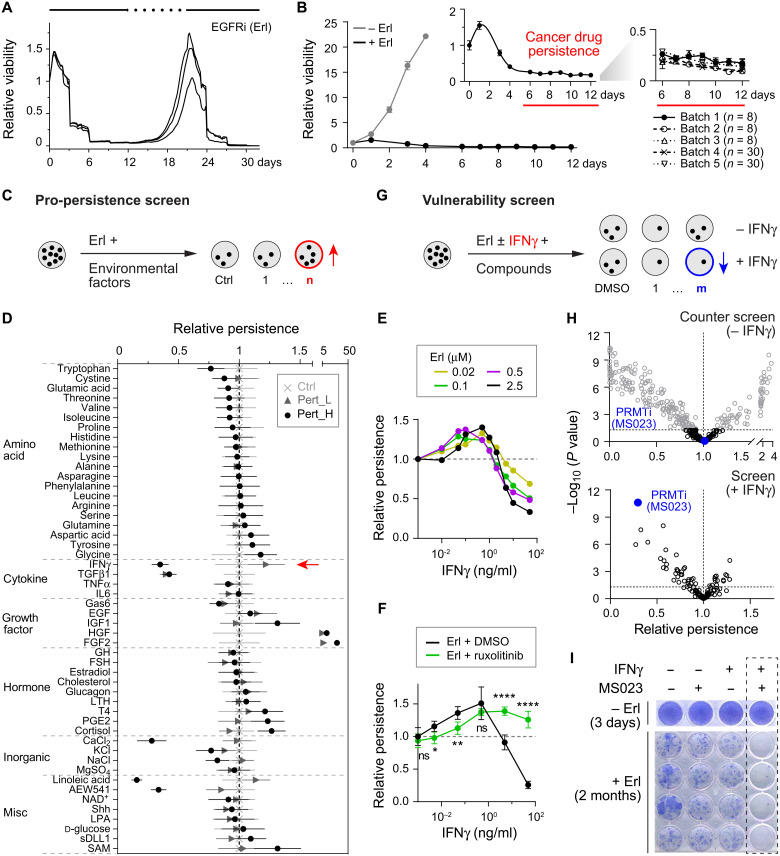
Sequential search for persistence modulators in PC9 cells. (**A**) Reversibility of cancer persistence [solid/dashed lines, on/off erlotinib (Erl)]. Relative cell viability normalized to day 0 (*n* = 3). (**B**) Left: Relative cell viability in − Erl (gray; *n* = 6) or + Erl (black; *n* = 8). Inset: Zoom-in of + Erl curve highlighting the emergence of persisters (red). Right: reproducibility across experimental batches. (**C**) Workflow of pro-persistence screen. Red: Pro-persistence factor. (**D**) Pro-persistence screen. Relative persistence normalized to controls (Ctrl). Pert_L/H, low/high-dose perturbations. Red arrow: IFNγ. TFGβ, transforming growth factor–β; TNFα, tumor necrosis factor–α; IL6, interleukin-6; GH, growth hormone; FSH, follicle stimulating hormone; LTH, luterotropic hormone; PGE2, prostaglandin E2; NAD^+^, nicotinamide adenine dinucleotide; LPA, lysophosphatidic acid; sDLL1, secreted extracellular domain of Notch receptor ligand DLL-1; SAM, S-(5’-adenosyl)-L-methionine. (**E**) Relative Erl persistence in ± IFNγ, normalized to − IFNγ (*n* = 6). (**F**) Relative Erl persistence in ± IFNγ + DMSO or ruxolitinib (200 nM), normalized to − IFNγ + DMSO (*n* = 3). (**G**) Workflow of persister vulnerability screen. Blue, context-dependent vulnerability. (**H**) Vulnerability screen. Relative persistence normalized to DMSO controls horizontal dashed line: *P* < 0.05. Compounds with no significant change in the counter screen (top) are shown (bottom). Blue, type I PRMT inhibitor MS023. (**I**) Crystal violet staining of cells in ± Erl ± IFNγ (0.5 ng/ml) ± MS023 (0.5 μM). Dashed box, antipersistence efficacy of MS023 in IFNγ. (A to I) Erl: 2.5 μM unless otherwise indicated. Error bar: SD. Unpaired, two-tailed *t* tests: ns, *P* > 0.05; *P ≤ 0.05; **P ≤ 0.01; ****P ≤ 0.0001.

We made use of the well-established persister model of PC9 lung cancer cells with epidermal growth factor receptor inhibitor (EGFRi) treatment (Fig. 1A) (*1*–*3*). A high dose of the EGFRi erlotinib (Erl) (>100-fold half maximal inhibitory concentration) causes marked cell death in the first several days (Fig. 1B, left); after ~1 week, a plateau phase is reached in which a small fraction of EGFRi-tolerant persister cells remains viable (Fig. 1B, inset of left). Traditional viability measurements (comparing drug-treated to nontreated conditions) are poorly suited to investigate changes to the small fraction of persisters that survive the initial treatment. Thus, we measured relative persistence (comparing perturbed to nonperturbed persister size in drug-treated conditions) to identify mechanisms and vulnerabilities that alter the emergence of persisters. We established the ability to perform reproducible, week-long persister assays at scale (Fig. 1B, right), and this allowed us to assess changes in the size of the small populations of persisters across perturbations.

### Sequential search for modulators of persistence

We first screened for cell signaling perturbations that promote persistence. Fifty extracellular factors found in tumor microenvironments were selected to alter a variety of intracellular signaling pathways (table S1). Cancer cells were pretreated with the perturbations and then with the EGFRi (Fig. 1C). Relative persistence was assessed by comparing the mean number of surviving persisters from perturbed versus nonperturbed conditions (Materials and Methods). The screen successfully captured a previously identified pro-persistence mechanism mediated by insulin-like growth factor 1 receptor (IGF1R) signaling (*1*) (IGF1 promoted and the IGF1R inhibitor AEW541 reduced the number of persisters; Fig. 1D). As expected, the growth factors HGF (hepatocyte growth factor) and FGF2 (fibroblast growth factor) significantly promoted the emergence of persisters (table S1) due to their ability to bypass EGFR signaling (*10*–*12*). We also identified conditions with uncharacterized pro-persistence functions.

Among the perturbations that promoted persistence, we chose to focus on the cytokine interferon-γ (IFNγ), as its effects were particularly intriguing. First, we observed that IFNγ both promoted (at a low dose) and mitigated (at a high dose) persistence (Fig. 1D; IFNγ did not markedly affect cell proliferation in the absence of EGFRi; fig. S1A and table S2). While IFNγ is classically reported as an anticancer cytokine (*13*–*16*), it was also reported to have a protective role for cancer in the context of radiation-induced genotoxicity (*17*–*19*). Second, recent literature reported that EGFRi treatment can elicit an IFNγ response gene expression signature in persisters (*20*, *21*), providing support for a connection between IFNγ signaling and persistence. Third, we observed this paradoxical pro- and antipersistence effect of IFNγ more generally across EGFRi concentrations (Fig. 1E), diverse apoptosis inducers (fig. S1B, left), and multiple persister models (fig. S1B, right). Last, attenuating IFNγ signaling through inhibition of Janus kinase 1/2 (JAK1/2) activities blocked the IFNγ-mediated antipersistence but not pro-persistence effects (Fig. 1F and fig. S1C).

We next searched for actionable vulnerabilities that reduce IFNγ-mediated persistence. About 100 small-molecule compounds were selected to perturb diverse biological processes, including cell signal transduction, protein translation, cellular metabolism, and epigenetic state (table S3). Cells were treated with EGFRi and compounds either in the absence or presence of IFNγ (Fig. 1G). Relative persistence was assessed by comparing the mean number of surviving persisters from compound-perturbed versus nonperturbed conditions (Materials and Methods). We found that the type I protein arginine methyltransferase (PRMT) inhibitor (PRMTi) MS023 (*22*) potently eliminated persisters in the presence of IFNγ yet had no effect in the absence of IFNγ (Fig. 1H and table S4). PRMTi’s efficacy was evident across a range of IFNγ concentrations based on measures of relative persistence (fig. S1D). (We note that this efficacy on persisters would have likely been missed using standard measures of relative viability; fig. S1E.) A long-term assay showed that the ability of PRMTi to eliminate persister survival/regrowth was both durable and specific to the IFNγ-induced cell signaling context (Fig. 1I). Together, our sequential screens identified matched roles for IFNγ and type I PRMTs in modulating positively and negatively (respectively) the emergence of cancer persisters.

### Pro- and antipersistence effects of IFNγ and type I PRMT inhibition depend on STAT1

**Fig. 2.**
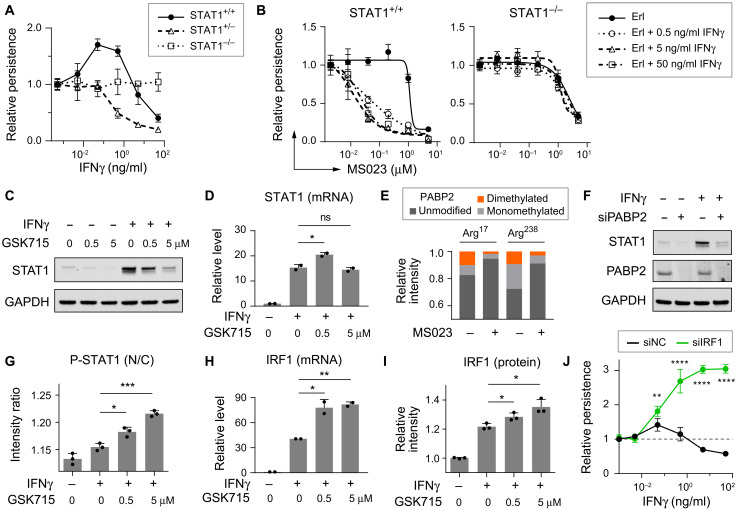
IFNγ’s pro-persistence and PRMTi’s antipersistence effects in PC9 cells depend on STAT1. (**A**) Relative Erl persistence of *STAT1*^+/+^, *STAT1*^+/−^, or *STAT1*^−/−^ cells in ± IFNγ, normalized to − IFNγ (*n* = 5). (**B**) Relative Erl persistence of STAT1^+/+^ (left) or STAT1^−/−^ (right) cells in ± MS023 ± IFNγ, normalized to − MS023 ± IFNγ (*n* = 3). STAT1 protein level (**C**) and mRNA level (**D**) of cells in ± GSK3368715 (GSK715) for 3 days, followed by ± IFNγ (5 ng/ml) for 1 day. (**E**) Relative intensity of unmodified, monomethylated, and dimethylated PABP2 peaks in mass spectrometry with cells in ± MS023 (5 μM) for 3 days. (**F**) STAT1 protein level of cells with ± PABP2 knockdown (siPABP2) for 3 days, followed by ± IFNγ (5 ng/ml) for 1 day. (**G**) P-STAT1 intensity ratio (N/C: nuclear versus whole cell) of cells in ± GSK715 for 3 days, followed by ± IFNγ (2 ng/ml) for 1 day. Shown are averages of single cell results in replicate wells (*n* = 3; >1000 cells per well). IRF1 mRNA level (**H**) and protein level (**I**) of cells in the same treatment as in (D) and (G), respectively. IRF1 intensity in whole cell was measured. (**J**) Relative Erl persistence of cells with (siIRF1) or without (siNC) knockdown for 2 days, followed by persister assays in ± IFNγ; normalized to − IFNγ with siNC (*n* = 5). (A to J) Erl: 2.5 μM. Error bar: SD. Unpaired, two-tailed *t* tests: ns, *P* > 0.05; *P ≤ 0.05; **P ≤ 0.01; ***P ≤ 0.001; ****P ≤ 0.0001. (C and F) Loading controls: Glyceraldehyde-3-phosphate dehydrogenase (GAPDH).

What connects IFNγ signaling and type I PRMTs in cancer persistence? We did not observe a change to the activities of classic EGFR bypass signaling components under cotreatment of EGFRi and different doses of IFNγ (fig. S2A) (*23*). Thus, the pro-persistence effect of IFNγ was unlikely to depend on EGFR bypass signaling. We next examined the IFNγ response in the PC9 persister model. We confirmed activation of the canonical IFNγ signaling pathway, represented by rapid phosphorylation of STAT1, subsequent up-regulation of interferon regulatory factor 1 (IRF1), and increased production of STAT1 itself (fig. S2B). Further, under both EGFRi-present or EGFRi-absent conditions, STAT1 expression was induced by IFNγ across the concentration range that elicited pro- and antipersistence effects (fig. S2C). We generated *STAT1* knockout cells (fig. S2, D and E) and found that partial deletion of *STAT1* (*STAT1^+/−^*) eliminated the pro-persistence and enhanced the antipersistence effects of IFNγ, while complete deletion of *STAT1* (*STAT1^−/−^*) eliminated both the pro- and antipersistence effects of IFNγ (Fig. 2A and fig. S2F). Thus, IFNγ’s ability to modulate persistence is completely dependent on STAT1. Third, we found that the antipersistence efficacy of PRMTi also depends on STAT1. Pan–type I PRMT inhibitors [MS023 or GSK3368715 (*24*)] reduced persisters starting from low-nanomolar concentrations (Fig. 2B, left, and fig. S2G, left). This reduction was specific to the IFNγ context and was lost in *STAT1*^−/−^ cells (Fig. 2B, right, and fig. S2G, right). Further, in month-long assays, type I PRMT inhibitors did not alter the survival and/or regrowth of persisters in *STAT1*^−/−^ cells regardless of IFNγ stimulation (fig. S2H). Together, our data show that modulation of persistence by IFNγ and PRMTi depends on STAT1.

### Mechanistic connection between STAT1, type I PRMT inhibition, and persistence

Type I PRMTs methylate numerous substrates (*25*–*27*); yet, while STAT1 has been controversially suggested to be a direct substrate of type I PRMTs (*28*, *29*), we did not observe direct STAT1 methylation in our system. We investigated whether PRMTi alters IFNγ-induced STAT1 expression or activity. First, we found that PRMTi decreased STAT1 protein levels (Fig. 2C and fig. S3A) but not mRNA expression levels (Fig. 2D and fig. S3B). Further, inhibition of proteasome or lysosome function did not rescue STAT1 reduction by PRMTi (fig. S3C). Thus, we reasoned that PRMTi-reduced STAT1 protein expression is likely due to decreased protein production rather than increased protein degradation. Type I PRMTs have been previously shown to regulate RNA processing via directly methylating RNA binding proteins (*25*). We found that PRMTi can decrease methylation of poly(A) binding protein 2 (PABP2) (Fig. 2E and replicate fig. S3, D and E), a poly(A)-binding protein and direct substrate of type I PRMTs (*30*). Further, PABP2 can regulate STAT1 protein expression. Knockdown of PABP2, but not the other two PABP isoforms PABP1 or PABP4, markedly reduced STAT1 protein levels (Fig. 2F and fig. S3F). Arginine methylation of PABP2 has been reported to mediate PABP2’s RNA processing function by regulating its self-association (*31*). These results show that PRMTi decreases STAT1 protein expression, likely by preventing the methylation of PABP2, which, in turn, reduces persistence.

Second, although PRMTi led to decreased total STAT1 protein expression, the nuclear fraction of phosphorylated STAT1 (P-STAT1) increased (Fig. 2G and fig. S3G). Further studies showed that PRMTi also increased mRNA and protein expression of IRF1, a primary target gene of P-STAT1 (Fig. 2, H and I, and fig. S3, H and I). Knockdown of IRF1 significantly increased persistence (Fig. 2J), indicating it as an antipersistence factor. Together, these results suggest that PRMTi can modulate persistence via decreasing pro-persistence STAT1-mediated signals (Fig. 2A) and increasing antipersistence P-STAT1–mediated signals (Fig. 1F).

### Type I PRMT inhibition reduces persistence with STAT1-high cancer cells

**Fig. 3.**
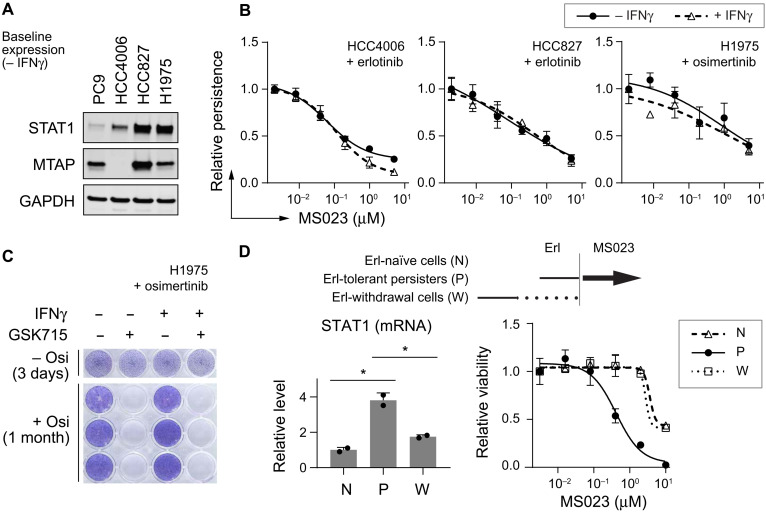
Type I PRMT inhibition reduces EGFRi persistence with STAT1-high cancer cells. (**A**) Baseline protein levels of NSCLC cells without IFNγ stimulation. Loading control: GAPDH. (**B**) Relative persistence of endogenously STAT1-high cells with EGFRi ± MS023 ± IFNγ (2 ng/ml), normalized to − MS023 ± IFNγ (*n* = 3). Erlotinib, 0.5 μM; osimertinib, 50 nM. No significant differences between ± IFNγ curves in each of the three models (two-tailed, paired *t* test; *P* > 0.05). (**C**) Crystal violet staining of H1975 cells in ± osimertinib (Osi, 100 nM) ± IFNγ (0.5 ng/ml) ± GSK715 (0.5 μM). (**D**) Effects of type I PRMT inhibition as a sequential treatment in PC9 cells. Top: Treatment schedules. Solid lines, + Erl (2.5 μM) for 6 days; dotted line, − Erl for 12 days; arrow, +MS023 for 9 days. Bottom left: STAT1 mRNA levels, normalized to Erl-naïve cells (*n* = 2). Unpaired, two-tailed *t* tests: *P ≤ 0.05. Bottom right: relative viability of cells, normalized to no MS023 controls (*n* = 5). (B and D) Error bar: SD.

Given the functional connection between STAT1 and type I PRMTs in modulating cancer persistence, we hypothesized that PRMTi could be effective in reducing persisters even without exogenous IFNγ stimulation. We tested whether PRMTi could reduce persisters from cancer cells with high endogenous STAT1 expression. In comparison to PC9 cells, the *EGFR* mutant non–small cell lung cancer (NSCLC) cells HCC4006, HCC827, and H1975 have higher levels of baseline STAT1 expression (Fig. 3A). Nevertheless, STAT1 levels in these models can be further induced by IFNγ (fig. S4, A and B). We established persistence assays with these cell lines using first- and third-generation EGFRi erlotinib and osimertinib. PRMTi diminished the emergence of persisters in all three models, regardless of extrinsic IFNγ stimulation (Fig. 3B and fig. S4C). PRMTi reduced persistence regardless of the cancer model’s S-methyl-5′-thioadenosine phosphorylase (MTAP) status [a biomarker for sensitivity to PRMTi monotherapy (*24*); Fig. 3A]. Consistently, using the H1975-osimertinib model, we observed durable potency of PRMTi to eliminate the survival and/or regrowth of persisters without the addition of extrinsic IFNγ (Fig. 3C).

We also tested whether PRMTi could be effective in reducing cancer regrowth during off-treatment periods in PC9 cells (Fig. 3D, top). This was motivated by recent observations that STAT1 expression can be increased by various cancer drugs (*20*, *21*, *32*). We confirmed that STAT1 expression increased after EGFRi treatment (Fig. 3D, bottom left, N versus P; and fig. S4D). Further, we found that immediate PRMTi treatment after removal of the EGFRi strongly reduced the proliferation of persisters (Fig. 3D, bottom right, P; and fig. S4E, P). This cellular efficacy is seen at submicromolar concentrations of PRMTi, which has no effect on the EGFRi-untreated cells. Consistent with the reversibility of the persister state, we observed that STAT1 expression decreased in cells that had been removed from EGFRi treatment (Fig. 3D, bottom left, W versus P). Further, we found that PRMTi treatment showed no specific efficacy to cells after a week-long withdrawal from EGFRi (Fig. 3D, bottom right, W; and fig. S4E, W). Thus, PRMTi specifically targets STAT1-high persisters rather than regrown STAT1-low cancer cells.

## DISCUSSION

Here, we demonstrate a strategy to identify and connect mechanisms of persistence with their vulnerabilities. Our discovery of IFNγ/STAT1-mediated persistence and its abrogation through type I PRMT inhibition relied on several key insights. First, changes to the small drug-tolerant persister population, rather than the bulk drug-sensitive population, are the functionally relevant readouts for investigating persister mechanisms. In combination with the EGFRi, type I PRMT inhibitors markedly decrease the emergence of persisters (Fig. 2B) yet have barely detectable effects on the bulk population (fig. S1E). Second, perturbations of cells with extracellular factors can help to identify intracellular signal transducers that promote persistence. The numerous molecular differences between persisters and their treatment-naïve counterparts [including epigenetic, transcriptional, translational, and metabolic alterations (*20*, *33*–*43*)] make it challenging to identify causal changes that lead to persistence, let alone targetable vulnerabilities. Third, the identification of pro-persistence signal transducers enables both literature-based and less targeted searches for vulnerabilities. Genetic knockdown of known IFNγ-STAT1 pathway components identified several regulators that could decrease overall levels of persistence, but none completely eliminated the characteristic increase of persistence at low IFNγ concentrations (fig. S5, A and B). Our broader screen provided a complementary approach to identify vulnerabilities beyond the immediately implicated pathway.

Our study found that net persistence levels reflected a balance between pro- and antisurvival signals transduced by STAT1. We found that inhibiting P-STAT1 promotes IFNγ-modulated persistence (Fig. 1F) while reducing STAT1 protein levels eliminates the pro-persistence effects of IFNγ (Fig. 2A). Further, we found that type I PRMT inhibition modulated STAT1 composition by enhancing activities of P-STAT1 (Fig. 2G) and decreasing protein levels of STAT1 (Fig. 2C). This is consistent with reported anti–cell survival effects of P-STAT1 versus prosurvival effects of the de novo–synthesized, largely unphosphorylated STAT1 (*44*–*47*).

This study motivates future investigations. These include identifying persister bypass mechanisms to type I PRMT inhibition, searching for other persister contexts (beyond IFNγ/STAT1 signaling) in which type I PRMTs are vulnerabilities and expanding the search for pro- and antipersistence signals. Our work lays a general foundation for exploring the landscape of mechanisms and vulnerabilities of cancer persisters.

## MATERIALS AND METHODS

### Cell lines and chemicals

The PC9, HCC827, HCC4006, H1975, and HCC2935 lung cancer cell lines were gifts from the John Minna Lab (University of Texas Southwestern Medical Center). The MGH119 cell line, derived from a treatment-naïve lung cancer patient with *EGFR* mutation, was a gift from Aaron Hata Laboratory (Massachusetts General Hospital). Cells were cultured in RPMI 1640 (Gibco #11835-030), unless otherwise indicated, supplemented with 5% heat-inactivated fetal bovine serum (Gemini #100-106) and 1× antibiotic-antimycotic (Gibco #15240-062) at 37°C, 5% CO_2_, and 90% humidity. Cell lines were tested by short tandem repeat fingerprinting to confirm their identities, and all cells were tested negative for mycoplasma. Erlotinib (S1023), gefitinib (S1025), osimertinib (S7297), olaparib (S1060), PAC-1 (S2738), MS023 (S8112), ruxolitinib (S1378), and bortezomib (S1013) were purchased from Selleck Chemicals. GSK3368715 was purchased from WuXi AppTec. Cycloheximide (#18079) and ammonium chloride (#254134) were purchased from Sigma-Aldrich. Recombinant human IFNγ (#300-02) and TNF-related apoptosis-inducing ligand (TRAIL) (#310-04) were purchased from PeproTech. Screening reagents are listed in tables S1 and S3.

### Persister assays

For the persister reversibility experiment in Fig. 1A, PC9 Histone H2B-mCherry cells (50,000 cells per well, 24-well plate) were monitored over time using the IncuCyte S3 automated imaging system (Sartorius). Phase and red channel images were taken every 4 hours (whole well imaging, 4× objective). Cell numbers per well and time point were extracted using built-in IncuCyte analysis software and normalized to day 0. For time course experiments in Fig. 1B, phase images of PC9 cells (96-well plate) were taken at each time point. Cell confluence (− Erl) or cell numbers (+ Erl) were normalized to day 0. For short-term persister emergence experiments, cells (1000 to 5000 per well, 96-well plate) were treated from the second day after seeding, and treatments were replenished every 3 days. Persistence levels were measured by Cell TiterGlo assay (Promega, G9243) at the end point (typically 6 days, unless otherwise indicated). For long-term persister regrowth experiments, cells (30,000 to 50,000 per well, 24-well plate) were treated from the second day after seeding, and treatments were replenished every week. At end points, cells were fixed in ice-cold methanol and stained with crystal violet (0.5%, w/v). Cells in the absence of EGFRi were fixed and stained before reaching complete confluence.

### Pro-persistence screen

PC9 cells were seeded into 96-well plates at 1000 cells per well. On the second day, cells were pulse perturbed with screen factors (table S1) for 2 hours and then treated with 2.5 μM Erl for 9 days. Erl-containing media were replenished every 3 days. At the end point, cells were stained with Hoechst 33342 (Thermo Fisher Scientific #H3570) and imaged for cell counting. Each perturbation and its corresponding nonperturbed control were assayed in the same plate with 15 technical replicates. Perturbations of interest were tested in independent experiments for confirmation. Relative persistence = (number of persisters in the perturbated or nonperturbed condition)/(mean number of persisters in the nonperturbed control condition). For cell viability tests in the absence of Erl, cells were pulse perturbed with screen factors (table S2) for 2 hours and then replenished with fresh media. Cell viability was measured by Cell TiterGlo assay at day 4.

### Vulnerability screen

PC9 cells were seeded into 96-well plates at 1000 cells per well. The following day (day 0), Erl (2.5 μM), IFNγ (±5 ng/ml), and compounds (table S3) were added. On day 3, the medium was changed (Erl-only), and persister viability was measured by Cell TiterGlo assay on day 6. Each perturbation has its own nonperturbed dimethyl sulfoxide (DMSO) control in the same plate (10 technical replicates). Multiple doses of compounds were tested, and perturbations of interest were further tested in independent experiments for confirmation. Relative persistence = (viability of persister cells in the perturbed condition)/(viability of persister cells in the nonperturbed condition) (table S4).

### Pro-persistence IFNγ concentrations

For PC9 cells, the pro-persistence range of IFNγ can depend on the treatment duration. We observed that IFNγ promotes persistence at 5 ng/ml in the initial pro-persistence screen (2-hour pulse perturbation of IFNγ before Erl) and the initial vulnerability screen (3-day Erl ± IFNγ followed by 3-day Erl). In subsequent experiments (6-day Erl ± IFNγ), IFNγ reproducibly promotes persistence between 0.05 and 0.5 ng/ml.

### CRISPR-mediated STAT1 deletion

PC9 *STAT1* knockout pool populations were generated by Synthego (guide sequence: TGTAAATGATCATAGACATC). Clonal populations were generated by limiting dilution and clonal expansion, and genomic DNA was extracted for genotyping (GeneWiz). Sequencing primers were 5′-TGAGGTTTCCCGGAGAATATAA-3′ (forward) and 5′-CTCCCACTTCTTGGGGCTAT-3′ (reverse). Sanger sequencing data were analyzed to determine editing efficiency (inference of CRISPR edits software, Synthego). Both homozygous and heterozygous *STAT1* knockout cells were obtained, and their *STAT1* deletion status was further validated by Western blot.

### RNA interference

For knockdown experiments, cells were transfected with small interfering RNAs (siRNAs; 25 nM) using Lipofectamine 3000 (Invitrogen) for 2 days before proceeding to persister assays or Western blot. ON-TARGET plus Human siRNA-SMARTpools (Dharmacon) used in the study are non-targeting control pool (D-001810-10), STAT1 (L-003543-00), IFNGR1 (L-011057-00), IFNGR2 (L-012713-00), JAK1 (L-003145-00), JAK2 (L-003146-00), IRF1 (L-011704-00), SOCS1 (L-011511-00), PIAS1 (L-008167-00), TCPTP (L-008969-00), ISG15 (L-004235-03), PABP1 (L-019598-00), PABP2 (L-011803-00), and PABP4 (L-011528-01).

### RNA isolation and reverse transcription qPCR

Total RNA was extracted from cells using the RNeasy Plus Mini Kit (Qiagen). The iScript Reverse Transcription Supermix (Bio-Rad) was used for cDNA synthesis according to the manufacturer’s instructions. Following the reverse transcription, mRNA abundance was determined in duplicates by quantitative polymerase chain reaction (qPCR) using SsoAdvanced Universal SYBR Green Supermix (Bio-Rad). Data were analyzed by the threshold cycle (*C*_t_) comparative method, and *ACTB* gene was used as the internal control. Primers used are as follows: ACTB, 5′-TCCCTGGAGAAGAGCTACG-3′ (forward) and 5′-GTAGTTTCGTGGATGCCACA-3′ (reverse); STAT1, 5′-GTTCAACATTTTGGGCACGCAC-3′ (forward) and 5′-AGGACCCTCATTCGTTCTGGTG-3′ (reverse); IRF1, 5′-TGGGACATCAACAAGGATGCCT-3′ (forward) and 5′-CACCTCCTCGATATCTGGCAGG-3′ (reverse); IFNGR1, 5′-GGAGACGAGCAGGAAGTCGATT-3′ (forward) and 5′-ACTGGAATCGCTAACTGGCACT-3′ (reverse); IFNGR2, 5′-GTCGGGCCTCCAGAAAACATTG-3′ (forward) and 5′-GCTTCTGAAAGGGCCTTTGACC-3′ (reverse); JAK1, 5′-CAAATCGCACCATCACCGTTGA-3′ (forward) and 5′-GCCACACTGACTGCTCATTGTC-3′ (reverse); JAK2, 5′-GATAGGTGCCCTGGGGTTTTCT-3′ (forward) and 5′-CCCAGTGTTGTCCTGTAGAGGG-3′ (reverse); ISG15, 5′-CTGAGAGGCAGCGAACTCATCT-3′ (forward) and 5′-CCTTCAGCTCTGACACCGACAT-3′ (reverse); TCPTP, 5′-GCAGCAGAAGACCAAAGCAGTT-3′ (forward) and 5′-ATCTGTTGGCCAGTACTGTGCA-3′ (reverse); PIAS1, 5′-CATCAGCTCTTCACCCAGTCCA-3′ (forward) and 5′-TTTGCTGCACTTGTTGTGGTGT-3′ (reverse); SOCS1, 5′-CTGTATCTGGAGCCAGGACCTG-3′ (forward) and 5′-CAACCCCTGGTTTGTGCAAAGA-3′ (reverse); PRMT1, 5′-ACTGGTGGGAGAACGTGTATGG-3′ (forward) and 5′-GGAGGTGAAGGTCAGGTCTTCC-3′ (reverse); PRMT4, 5′-ATAATGACCGTGTGGCTGTCCA-3′ (forward) and 5′-TGGGCCACAATACTGATGTCGT-3′ (reverse); and PRMT6, 5′-GAGCAAGACACGGACGTTTCAG-3′ (forward) and 5′-CTCCTCCTGGTCTCCCACTTTG-3′ (reverse).

### Immunofluorescence assay

Experiments were performed in 96-well plates. Cells were washed 1× with warm Dulbecco’s phosphate-buffered saline (PBS) and then fixed with 4% paraformaldehyde in PBS for 15 min at room temperature. Cells were then washed with PBS and permeabilized with 0.5% Triton X-100 in PBS at room temperature for 10 min. Cells were washed, blocked with 3% bovine serum albumin (BSA) in PBS for 30 min, and then incubated in primary antibody in antibody buffer (PBS with 0.3% Triton X-100 and 1% BSA) overnight at 4°C. The next day, cells were washed and incubated with secondary antibodies and Hoechst 33342 (2 μg/ml; Thermo Fisher Scientific #H3570) in antibody buffer for 2 hours at room temperature. After this, cells were washed with PBS and imaged in TBS-T [0.1% Tween 20 in 1× tris-buffered saline (pH 7.4)] on PerkinElmer Operetta High-Content Imaging System (25 fields per well, 20× objective, nonconfocal mode). The following antibodies were used: IRF1 (Cell Signaling Technology #8478), STAT1 (BD Biosciences #610186), and phospho-STAT1 (BD Biosciences #612133).

### Immunofluorescence image segmentation and quantification

Image analysis was performed using the PerkinElmer Harmony 4.9 software. Images were subject to flatfield correction (advanced setting), followed by a segmentation step to generate boundaries of objects (Hoechst-stained images for finding nuclei; antibody-stained images for finding cells). Nonborder cells were selected, and signal intensities were calculated for single cells. The quantification of single cells and well-level average (at least 1000 cells per well) were extracted. The measurements of three replicate wells were depicted.

### Western blot

Protein was denatured in Laemmli sample buffer (Bio-Rad), resolved on Mini-PROTEAN 4 to 20% gels (Bio-Rad) with tris/glycine/SDS buffer (Bio-Rad), and then transferred to 0.45-μm polyvinylidene difluoride membranes (Thermo Fisher Scientific). Blots were blocked in Odyssey blocking buffer (LI-COR) and followed by incubation with primary antibodies diluted in blocking buffer overnight at 4°C with agitations. Blots were washed thoroughly in TBS-T (0.1% Tween 20 in 1× tris-buffered saline), and secondary antibodies (IRDye, LI-COR) were applied with incubation at room temperature for 1 hour. Blots were scanned using LI-COR Odyssey imager. The following antibodies were purchased from Cell Signaling Technology: STAT1 (#14994), phospho-STAT1 (#9167), IRF1 (#8478), PABP2 (#14154), phospho-EGFR (#3777), EGFR (#4267), phospho-AKT (#4060), phospho-ERK (#5726), phospho-STAT3 (#9145), MTAP (#62756), and glyceraldehyde-3-phosphate dehydrogenase (GAPDH; #5174). PABP1 (ab6125) and PABP4 (ab241592) antibodies were purchased from Abcam.

### Mass spectrometry for PABP2 methylation

Cells were lysed with Pierce IP lysis buffer (Thermo Fisher Scientific) supplemented with Halt protease and phosphatase inhibitor cocktail (Thermo Fisher Scientific #78440). The supernatant of cell lysates was immunoprecipitated using the cross-linked PABP2 antibody–Protein A/G agarose beads (Pierce Crosslink IP kit). The affinity elute was subjected to SDS–polyacrylamide gel electrophoresis and stained with colloidal Coomassie blue (Thermo Fisher Scientific). Bands were excised at molecular weight of target protein and then subject to in-gel digestion by trypsin. Digested protein was analyzed by liquid chromatography–tandem mass spectrometry. Chromatography was performed using a NanoAcquity (Waters) LC system at a flow rate of 400 nl/min. A gradient of 2 to 25% B (acetonitrile and 0.1% formic acid) over 48 min was followed by a gradient up to 37% B over a further 8 min, before briefly increasing to 80% B and then returning to starting conditions. Eluent was introduced into a QExactive Plus (Thermo Fisher Scientific) mass spectrometer, where a “Top 10” method was used where cycles of the 10 most intense precursor peaks detected in each survey scan were selected for fragmentation analysis, before being added to an exclusion list so different peptides are selected in the following cycles. Raw data were converted into peak list files using PAVA and then analyzed using Protein Prospector v6.1. Spectra were searched against a concatenated database of all human entries in SwissProt and randomized versions to allow for false discovery rate estimation. Precursor and fragment mass tolerances were set at 20 parts per million. Modifications considered included methionine oxidation, pyroglutamate formation from peptide N-terminal glutamine, protein N-terminal methionine removal and/or acetylation, monomethylation of D, E, or R, and dimethylation of R. Quantification of methylation states was performed on the basis of the relative peak intensities of peptides containing different arginine methylation states of the same residue. The peptides quantified are MAAAAAAAAAAGAAGGR [mass/charge ratio (*m*/*z*) = 620.8^+2^, containing unmodified Arg^17^], MAAAAAAAAAAGAAGGR*GSGPGR (*m*/*z* = 589.3^+3^, containing methylated Arg^17^), MAAAAAAAAAAGAAGGR**GSGPGR (*m*/*z* = 594.0^+3^, containing dimethylated Arg^17^), TSLALDESLFR (*m*/*z* = 626.3^+2^, containing unmodified Arg^238^), TSLALDESLFR*GR (*m*/*z* = 493.6^+3^, containing methylated Arg^238^), and TSLALDESLFR**GR (*m*/*z* = 498.3^+3^, containing dimethylated Arg^238^). R* is the methylated arginine, and R** is the dimethylated arginine. As the arginine methylation inhibits trypsin cleavage at the modified residue, the peptide used for measuring the unmodified state was the one where trypsin cleaved after the relevant arginine residue; whereas for monomethylation and dimethylation states, the peptide contained a missed cleavage site.

### Statistical analyses

Statistical tests and graphing of data were performed with GraphPad Prism (version 7.01). For comparisons between PRMTi dose-response curves in + IFNγ and − IFNγ conditions in Fig. 3B, *P* values were calculated using paired, two-tailed *t* tests. For other experiments, *P* values were calculated using unpaired, two-tailed *t*-tests. The meanings of symbols are as follows: ns indicates *P* > 0.05, *P ≤ 0.05, **P ≤ 0.01, ***P ≤ 0.001, and ****P ≤ 0.0001.

## Notes

View/request a protocol for this paper from *Bio-protocol*.
