# Supplementary material for: Modulating environmental signals to reveal mechanisms and vulnerabilities of cancer persisters
Source: Sci Adv. 2022 Jan 28;8(4):eabi7711. doi: 10.1126/sciadv.abi7711 (PMC8797778; doi:10.1126/sciadv.abi7711)
Supplement: Supplementary file 1 — Figs. S1 to S5 [file sciadv.abi7711_sm.pdf]

Science Advances
AAAS

Supplementary Materials for

Modulating environmental signals to reveal mechanisms and vulnerabilities of cancer persisters

Xiaoxiao Sun, Jake M. Bieber, Heinz Hammerlindl, Robert J. Chalkley, Kathy H. Li, Alma L. Burlingame, Matthew P. Jacobson, Lani F. Wu*, Steven J. Altschuler*

*Corresponding author. Email: steven.altschuler@ucsf.edu (S.J.A.); lani.wu@ucsf.edu (L.F.W.)

Published 28 January 2022, *Sci. Adv.* **8**, eabi7711 (2022)
DOI: 10.1126/sciadv.abi7711

The PDF file includes:

Figs. S1 to S5

Other Supplementary Material for this manuscript includes the following:

Tables S1 to S4

figure S1

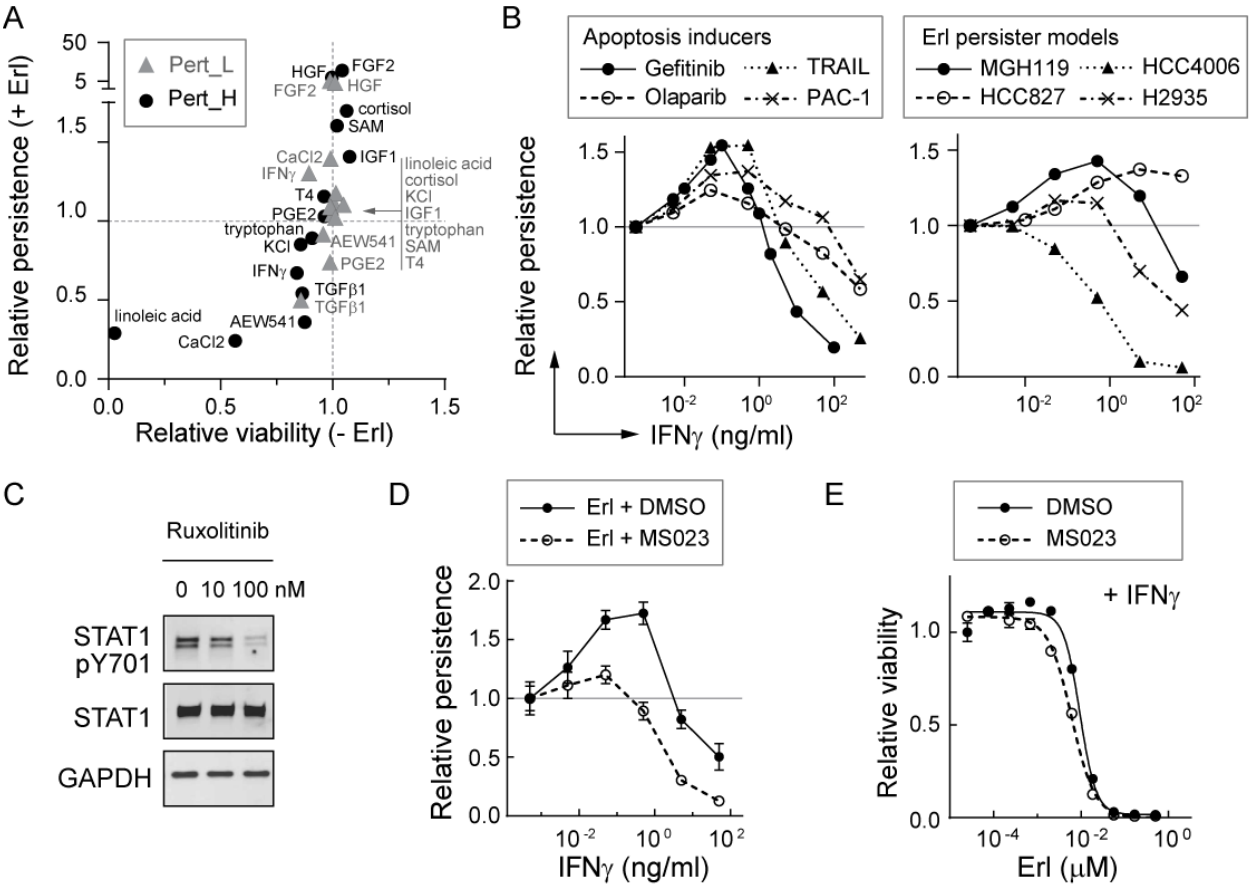

figure S1. IFNγ promotes and type I PRMT inhibition eliminates emergence of persisters. A. Follow-up of the pro-persistence screen. Pert_L: low dose of perturbations; Pert_H: high dose of perturbations. Relative persistence (y-axis): pre-perturbed PC9 cells in 2.5 μM Erl (normalized to non-perturbed condition; n=15). Relative viability (x-axis): pre-perturbed PC9 cells in the absence of Erl (normalized to non-perturbed condition; n=15). **B.** IFNγ dose response for persistence in additional models. Relative persistence: normalized to no IFNγ condition. Left: PC9 cells treated with apoptosis inducers ± IFNγ for 6 days. Gefitinib (EGFRi), 1 μM; Olaparib (PARP inhibitor), 100 μM; TRAIL (death ligand), 100 ng/ml; PAC-1 (procaspase-3 activator), 1 μM. Shown are mean values (n=5-6). Right: EGFR mutant lung cancer cells treated with Erl ± IFNγ for 6 days. Erl concentrations: 2.5 μM for HCC827, HCC4006, and HCC2935; 10 nM for MGH119. Shown are mean values (n=3-5). **C.** Western blots showing effects of JAK1/2 inhibition. PC9 cells treated with 2.5 μM Erl + 50 ng/ml IFNγ + indicated amount of Ruxolitinib for 2 days. GAPDH used as a loading control. **D.** PC9 cells treated with 2.5 μM Erl ± 0.2 μM MS023 ± various concentrations of IFNγ for 6 days. Relative persistence: normalized to no IFNγ with each MS023 condition. Error bars: standard deviations (n=5). **E.** PC9 cells treated with 0.5 ng/ml IFNγ ± 0.5 μM MS023 ± various concentrations of Erl for 6 days. Relative viability: normalized to no MS023 and Erl condition. Error bars: standard deviations (n=3).

figure S2

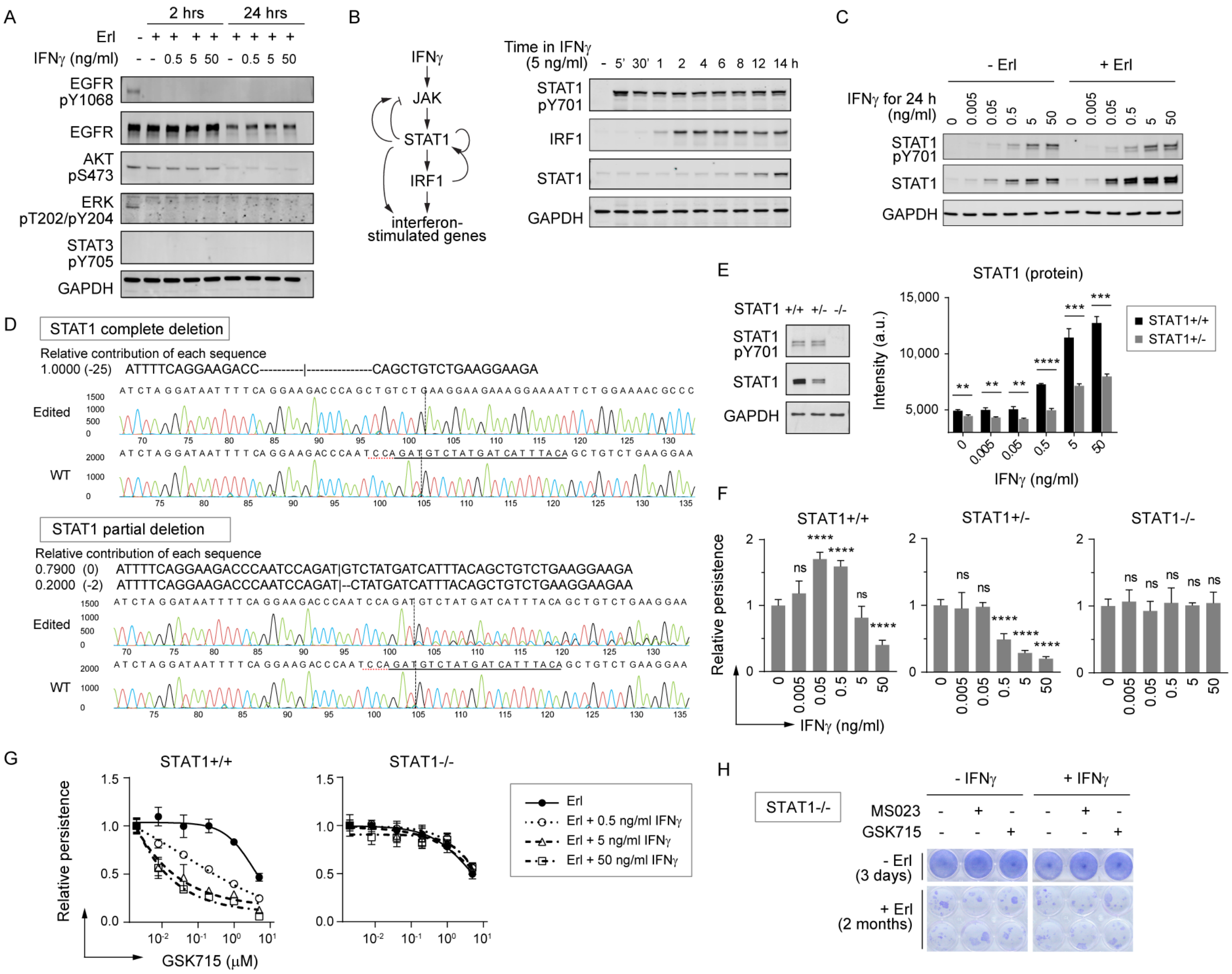

figure S2. IFNγ's pro-persistence and PRMTi's anti-persistence effects in PC9 cells depend on STAT1. A. Western blots showing effects of IFNγ on EGFR bypass signaling. Cells treated with ± 2.5 μM Erl ± indicated amount of IFNγ for 2 hours or 24 hours. GAPDH used as a loading control. **B.** Left: canonical IFNγ signaling pathway. Right: western blots showing time course of IFNγ-induced STAT1 pathway activation. Cells treated with 5 ng/ml IFNγ for the indicated time. GAPDH used as a loading control. **C.** Western blots showing dose response of IFNγ-induced STAT1 pathway activation. Cells treated with indicated amount of IFNγ for 24 hours in the absence (left) or presence (right) of 2.5 μM Erl. **D.** STAT1 knockout cells generated by CRISPR editing. Relative contribution of each sequence: proportion of cells with inferred sequence in the whole population of cells (parentheses: indel). Sequences in the region around the guide (black underline) and PAM site (red dashed underline) are shown in Sanger sequencing traces. Vertical line: the cut site. **E.** Left: western blots showing effects of STAT1 knockdowns. $STAT1^{+/+}$, $STAT1^{+/-}$, and $STAT1^{-/-}$ cells treated with 2.5 μM Erl + 0.5 ng/ml IFNγ for 2 days. GAPDH used as a loading control. Right: immunofluorescence data showing effects of heterozygous STAT1 knockdown on STAT1 protein levels. $STAT1^{+/+}$ and $STAT1^{+/-}$ cells treated with indicated amount of IFNγ for 1 day. Intensity of STAT1 protein: STAT1 mean intensity in the whole cell; shown are averages of single cell result for each well (>1000 cells per well). Error bars: standard deviations (n=3). Unpaired, two-tailed *t*-test: **, $p \leq 0.01$; ***, $p \leq 0.001$; ****, $p \leq 0.0001$. **F.** Statistical analysis of Fig 2A. IFNγ dose response on persistence of isogenic

STAT1$^{+/+}$, STAT1$^{+/-}$, or STAT1$^{-/-}$ PC9 cells with 2.5 μM Erl for 6 days. Relative persistence: normalized to no IFNγ condition with each cell line. Error bars: standard deviations (n=5). Unpaired, two-tailed *t*-test: indicated amount of IFNγ vs. no IFNγ (ns, $p > 0.05$; ****, $p \leq 0.0001$). **G.** GSK715 dose response on persistence. STAT1$^{+/+}$ (left) or STAT1$^{-/-}$ (right) cells treated with 2.5 μM Erl ± indicated amount of IFNγ ± various concentrations of GSK715 for 6 days. Relative persistence: normalized to no GSK715 with indicated amount of IFNγ. Error bars: standard deviations (n=3). **H.** Crystal violet staining of STAT1$^{-/-}$ cells in ± 2.5 μM Erl ± 0.5 μM MS023 or GSK715 for indicated time in the absence (left) or presence (right) of 0.5 ng/ml IFNγ.

figure S3

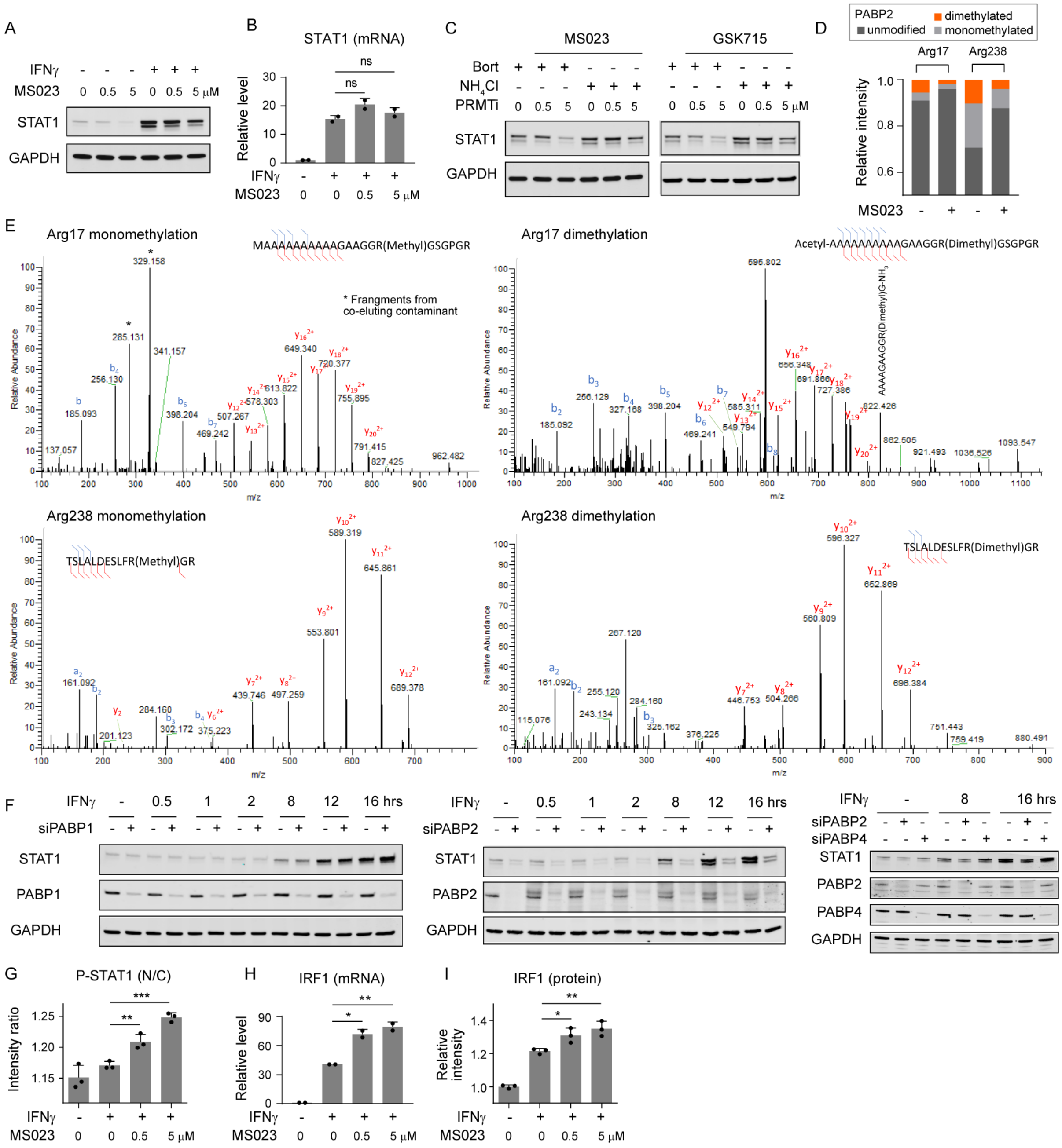

figure S3. Type I PRMT inhibition regulates STAT1 in PC9 cells. A-B. Effects of MS023 on STAT1 protein levels (**A**, western blot) and mRNA levels (**B**, qPCR). PC9 cells treated with or without indicated amount of MS023 for 3 days, followed by ± 5 ng/ml IFNγ stimulation for 1 day. **A.** GAPDH used as a loading control. **B.** Relative level: normalized to no IFNγ and MS023 condition. Error bars: standard deviations (n=2). **C.** Effects of proteasome inhibitor Bortezomib (Bort) or lysosomal activity inhibitor NH_4Cl on PRMTi-reduced STAT1. Cells treated with indicated amount of MS023 or GSK715 for 3 days, followed by 5 ng/ml IFNγ ± 10 nM Bort ± 10 mM NH_4Cl for 1 day. GAPDH used as a loading control. **D.** Biological replicates of mass spec data in Fig 2E. Cells treated with or without 5 μM MS023 for 3 days. Relative intensity for methylation of PABP2 at Arg17 and Arg238: proportion of intensities with unmodified,

monomethylated, and dimethylated peaks. **E.** Representative mass spectra of identified PABP2 peptides containing monomethylated and dimethylated Arg17 and Arg238. **F.** Western blots showing effects of PABP knockdowns on STAT1 protein levels. Cells incubated with scrambled siRNA, or siRNAs targeting PABP1, PABP2 or PABP4 for 2 days, followed by ± 5 ng/ml IFNγ stimulation for indicated time. GAPDH used as a loading control. **G.** Immunofluorescence data showing effects of MS023 on P-STAT1. Cells treated with or without indicated amount of MS023 for 3 days, followed by ± 2 ng/ml IFNγ stimulation for 1 day. Intensity ratio: P-STAT1 mean intensity in the nucleus vs. in the whole cell; shown are averages of single cell result in each well (>1000 cells per well). Error bars: standard deviations (n=3). **H-I.** Effects of MS023 on IRF1 mRNA levels (**H**, qPCR) and protein levels (**I**, immunofluorescence). **H.** Treatment: the same as in **B**. Relative level: normalized to no IFNγ and MS023 condition. **I.** Treatment: the same as in **G**. Relative intensity (normalized to no IFNγ and MS023 condition): average of IRF1 mean intensity in single cells in each well (>1000 cells per well). Error bars: standard deviations (n=3). Statistical analysis in **B** and **G-I**: unpaired, two-tailed *t*-test (ns, $p > 0.05$; *, $p \leq 0.05$; **, $p \leq 0.01$; ***, $p \leq 0.001$).

figure S4

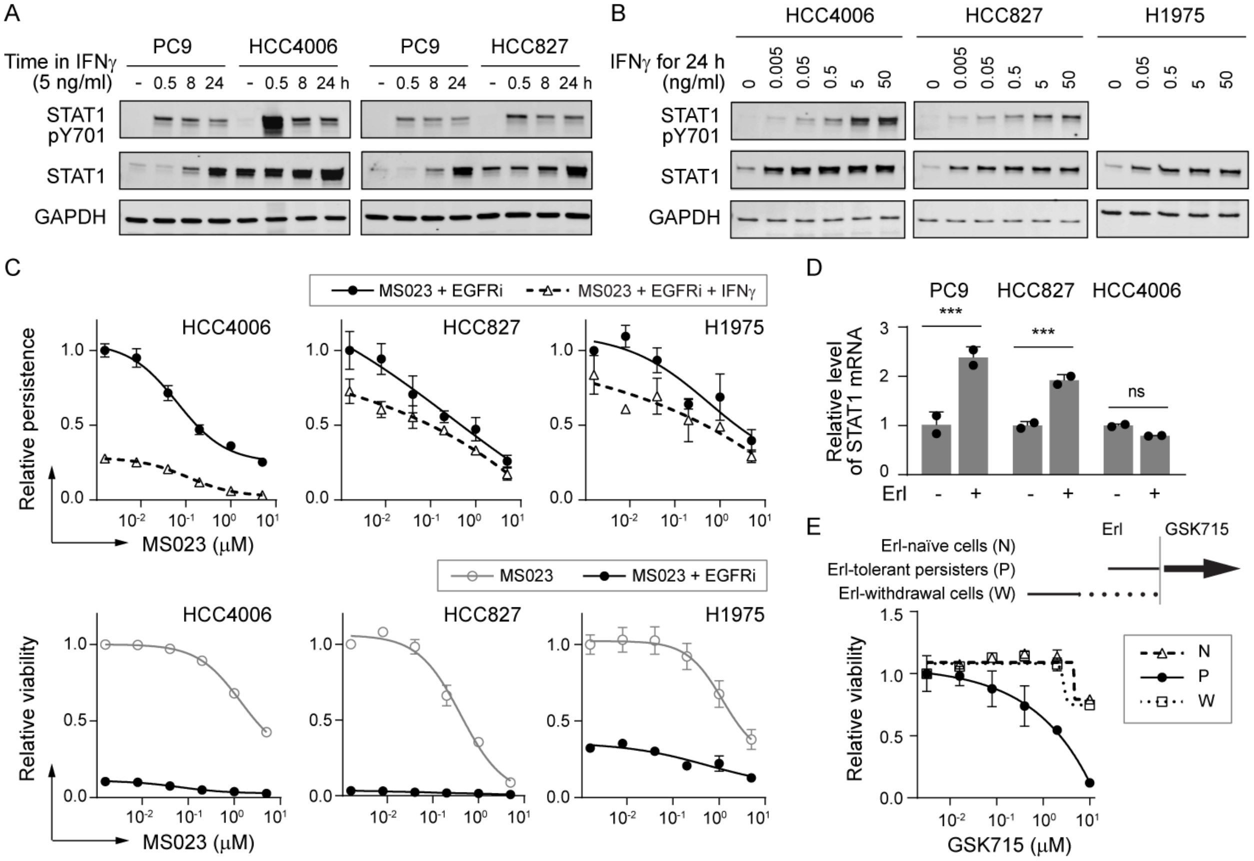

figure S4. Type I PRMT inhibition reduces EGFRi persistence in STAT1-high cancer cells. A. Western blots showing time course of IFNγ-induced STAT1 pathway activation. Cells treated with 5 ng/ml IFNγ for the indicated time. GAPDH used as a loading control. **B.** Western blots showing dose response of IFNγ-induced STAT1 pathway activation. Cells treated with indicated amount of IFNγ for 24 hours. **C.** Top: different normalization for Fig 3B. Relative persistence (normalized to no MS023 and IFNγ condition) of cells treated with ± MS023 + EGFRi ± 2 ng/ml IFNγ for 6 days. Bottom: relative viability (normalized to no MS023 and EGFRi condition) of cells treated with ± MS023 ± EGFRi for 6 days. EGFRi: 0.5 μM Erlotinib for HCC4006 and HCC827; 50 nM Osimertinib for H1975. Error bars: standard deviations (n=3). **D.** Relative level of STAT1 mRNA (normalized to no Erl) of cells in ± 2.5 μM Erl for 3 days. Error bars: standard deviations (n=2). Unpaired, two-tailed *t*-test: ns, $p > 0.05$; ***, $p \leq 0.001$. **E.** Effects of GSK715 as a sequential treatment in PC9 cells. Top: treatment schedules. Solid lines: 6 days with 2.5 μM Erl; dotted line: 12 days without Erl; arrow: 9 days with GSK715. Bottom: relative viability of cells (normalized to no GSK715 with each treatment schedule). Error bars: standard deviations (n=5).

figure S5

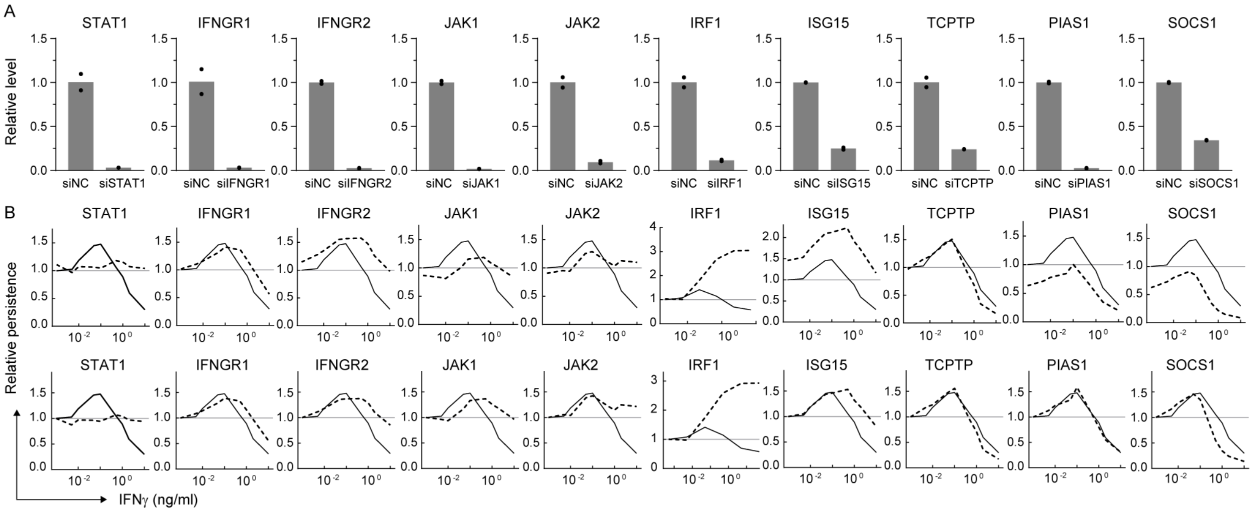

figure S5. Effects of canonical IFNγ pathway components on Erl persistence in PC9 cells. A. qPCR data showing efficiency of knockdowns along the canonical IFNγ pathway. Cells incubated with scrambled siRNA (siNC) or gene-targeted siRNAs for 1 day. Relative level of mRNA for the indicated gene: normalized to siNC (n=2). **B.** Effects of knockdowns on IFNγ-modulated persistence. Cells incubated with siNC (solid lines) or gene-targeted siRNAs (dashed lines) for 2 days, followed by co-treatment of 2.5 μM Erl ± IFNγ for 6 days. Relative persistence: solid lines, normalized to no IFNγ with siNC; dashed lines, normalized to no IFNγ with siNC (top) or no IFNγ with gene-targeted siRNAs (bottom). Shown are mean values (n=3-5).
